# Identification of 35 C-Type Lectins in the Oriental Armyworm, *Mythimna separata* (Walker)

**DOI:** 10.3390/insects12060559

**Published:** 2021-06-16

**Authors:** Hao Li, Fang-Fang Liu, Li-Qing Fu, Ze Liu, Wen-Ting Zhang, Qian Wang, Xiang-Jun Rao

**Affiliations:** 1 School of Plant Protection, Anhui Agricultural University, Hefei 230036, China; 19720142@ahau.edu.cn (H.L.); 19720147@ahau.edu.cn (F.-F.L.); 18720054@ahau.edu.cn (L.-Q.F.); llzz@ahau.edu.cn (Z.L.); 15103867@ahau.edu.cn (W.-T.Z.); 20720220@ahau.edu.cn (Q.W.); 2 Anhui Province Key Laboratory of Integrated Pest Management on Crops, Anhui Agricultural University, Hefei 230036, China

**Keywords:** *Mythimna separata*, C-type lectin, transcriptome

## Abstract

**Simple Summary:**

The oriental armyworm *Mythimna separata* is a lepidopteral agricultural pest that causes serious damage to many crops, such as maize, wheat, and sorghum. To control this pest, it is advisable to take comprehensive measures, including the use of chemical pesticides, microbial pesticides, and cultural practices. However, microbial pesticides (entomopathogens) can be eliminated by the insect immune system. C-type lectins (CTLs) are a family of pattern-recognition receptors that recognize carbohydrates and mediate immune responses. C-type lectins in the oriental armyworm have not yet been identified and characterized. In this study, a transcriptome of *M. separata* larvae was constructed and a total of 35 CTLs containing single or dual carbohydrate-recognition domains (CRDs) were identified from unigenes. Phylogenetic analyses, sequence alignments and structural predictions were performed. Gene expression profiles in different developmental stages, naïve larval tissues, and bacteria/fungi-challenged larvae were analyzed. Overall, our findings indicate that most dual-CRD CTLs are expressed in mid-late-stage larvae, pupae, and adults. Bacterial and fungal challenges can stimulate the expression of many CTLs in larval hemocytes, fat body, and midgut. Our data suggest the importance of CTLs in immune responses of *M. separata*.

**Abstract:**

Insect C-type lectins (CTLs) play vital roles in modulating humoral and cellular immune responses. The oriental armyworm, *Mythimna separata* (Walker) (Lepidoptera: Noctuidae) is a migratory pest that causes significant economic loss in agriculture. CTLs have not yet been systematically identified in *M. separata*. In this study, we first constructed a transcriptome of *M. separata* larvae, generating a total of 45,888 unigenes with an average length of 910 bp. Unigenes were functionally annotated in six databases: NR, GO, KEGG, Pfam, eggNOG, and Swiss-Prot. Unigenes were enriched in functional pathways, such as those of signal transduction, endocrine system, cellular community, and immune system. Thirty-five unigenes encoding C-type lectins were identified, including CTL-S1~CTL-S6 (single CRD) and IML-1~IML-29 (dual CRD). Phylogenetic analyses showed dramatic lineage-specific expansions of IMLs. Sequence alignment and structural modeling identified potential ligand-interacting residues. Real-time qPCR revealed that CTL-Ss mainly express in eggs and early stage larvae, while IMLs mainly express in mid-late-stage larvae, pupae, and adults. In naïve larvae, hemocytes, fat body, and epidermis are the major tissues that express CTLs. In larvae challenged by *Escherichia coli*, *Staphylococcus aureus*, or *Beauveria bassiana*, the expression of different CTLs was stimulated in hemocytes, fat body and midgut. The present study will help further explore functions of *M. separata* CTLs.

## 1. Introduction

Insects depend on the innate immune system to recognize and eliminate pathogens [1]. Germline-encoded pattern recognition receptors (PRRs) can recognize pathogen-associated molecular patterns (PAMPs) to trigger immune responses. Common PAMPs include bacterial lipopolysaccharide, lipoteichoic acid, peptidoglycans, fungal glucans, viral capsid, nucleic acids, and parasitic molecular patterns [2]. Numerous insect PRRs have been identified, such as C-type lectins (CTLs), peptidoglycan-recognition proteins (PGRPs), and β-1,3-glucan recognition proteins (βGRPs) [3,4,5,6].

CTLs are ubiquitous in plants, invertebrates, fungi, bacteria, and vertebrates. CTLs contain at least one carbohydrate-recognition domain (CRD) or C-type lectin-like domain (CTLD). Insect CTLs can be classified based on the domain architecture: CTL-S has a single CRD; immulectin (IML) has dual CRD; CTL-X has CRD and other motifs [7,8]. CTL-S and CTL-X are identified in many insect orders, while IMLs almost only exist in Lepidoptera [5]. Most CRDs consist of 110~130 amino acids and one to four Ca^2+^ binding sites. Ca^2+^ helps to maintain a stable protease-resistant structure. Ca^2+^ at site 2 mediates the recognition of carbohydrates through coordination bonds formed with hydroxyl groups on the sugar ring and amino acid side chains. CRDs with Glu–Pro–Asn (EPN) and Gln–Pro–Asp (QPD) motifs generally recognize mannose-type and galactose-type ligands, respectively. The differential arrangement of 3-OH and 4-OH on the pyranose ring is a major determinant in ligand preference [9]. In rat mannose-binding protein MBP-A, Glu^193^, Asn^205^, Asp^206^, Glu^185^, Asn^187^, Ca^2+^, and mannose form a ternary complex through a network of coordination and hydrogen bonds [10].

CTLs are involved in regulating humoral responses (phenoloxidase activation and antimicrobial peptide production) and cellular responses (encapsulation, nodulation, and phagocytosis). *Manduca sexta* immulectin-1 and immulectin-2 stimulate prophenol oxidase [11,12]. *M. sexta* immulectin-4 enhanced hemocyte encapsulation and melanization [13]. Two *Drosophila* CTLs can enhance encapsulation and melanization [14]. Knockdown of a CTL in *Tribolium castaneum* caused a significant decrease in antimicrobial peptides and transcription factors under lipopolysaccharide and peptidoglycan stimulation [15]. *Helicoverpa armigera* C-type lectin 7 can enhance hemocytes-mediated encapsulation and melanization [16]. *H. armigera* CTL14 depletion decreased the resistance to fungal challenge [17]. *Bombyx mori* lipopolysaccharide-binding protein participates in hemocyte nodule formation [18]. *B. mori* multibinding protein can trigger nodule reaction [19]. Some pathogens can subvert or use host CTLs to assist infection. The parasitoid, *Pteromalus puparum*, can suppress immune responses of the host, *Pieris rapae*, by silencing host CTL expression [20]. An *Aedes aegypti* C-type lectin (mosGCTL-1) facilitates West Nile Virus infection [21].

Caterpillars of the oriental armyworm feed on young seedlings or leaves of maize, wheat, sorghum, and millets. A large armyworm population may cause defoliation or damage corn cobs [22]. Using entomopathogens for pest control can efficiently prevent pesticide resistance [23]. Understanding the molecular interactions between entomopathogens and insects may help to develop new strategies for pest management [24]. Although a few immune factors in *M. separata* have been identified, less is known about its CTL family [25,26,27]. This study aims to construct a transcriptome of *M. separata* larvae and identify CTLs. In addition, the bioinformatic analyses and expression pattern assays will help to elucidate their roles in the development and immune system.

## 2. Materials and Methods

### 2.1. Insects and Microorganisms

*Mythimna separata* were collected in an experimental field at Anhui Agricultural University, Hefei, China. Larvae and adults were reared at 25 °C (photoperiod 12L:12D). Microorganisms used in the study (*Escherichia coli* DH5α, *Staphylococcus aureus*, and *Beauveria bassiana* ARSEF 2860) were kindly given by Dr. Erjun Ling from the Institute of Plant Physiology and Ecology, Shanghai, China.

### 2.2. RNA Sample Preparation, Library Construction, and Sequencing

Fourth instar larvae were frozen in liquid nitrogen and stored at −80 °C. Total RNA was isolated using the Trizol Reagent (Invitrogen Life Technologies, Carlsbad, CA, USA). RNA concentration was determined using a NanoDrop spectrophotometer (Thermo Scientific, Waltham, MA, USA). RNA quality and integrity were determined by RNA agarose gel electrophoresis and Agilent Bioanalyzer 2100 system. Three micrograms of RNA were used as input material for the RNA sample preparations. Sequencing libraries were generated using the TruSeq RNA Sample Preparation Kit (Illumina, San Diego, CA, USA). To select cDNA fragments of the preferred 200 bp in length, the library fragments were purified using the AMPure XP system (Beckman Coulter, Beverly, CA, USA). DNA fragments with ligated adaptor molecules on both ends were selectively enriched using Illumina PCR Primer Cocktail in a 15 cycle PCR reaction. Products were purified and quantified using the Agilent high sensitivity DNA assay on a Bioanalyzer 2100 system. The sequencing library was sequenced on a NovaSeq 6000 platform (Illumina) by Personal Biotechnology Co., Ltd (Nanjing, Jiangsu, China).

### 2.3. De Novo Transcriptome Analysis Flow

Raw data were filtered to remove low-quality reads using Cutadapt v2.7 to generate clean data (>10 bp overlap: AGATCGGAAG; 20% base error rate was allowed) [28]. Trinity v2.5.1 with the default setting was used to montage clean reads to generate transcript sequence files [29]. The longest transcript of each gene (Unigene) was extracted as the representative sequence of the gene. Databases used in gene annotation include NR (NCBI non-redundant protein sequences), GO (Gene Ontology), KEGG (Kyoto Encyclopedia of Genes and Genome), eggNOG (evolutionary genealogy of genes: Non-supervised Orthologous Groups), Swiss-Prot, and Pfam.

### 2.4. Identification of CTLs from the Transcriptome and Bioinformatic Analyses

To identify unigenes that encode CTLs, other annotated CTLs (*Manduca sexta*, *Bombyx mori*, *Spodoptera litura*, *Helicoverpa armigera*, *Apis mellifera*) were used as queries for tblastn searches [30]. Putative CTL sequences were confirmed by searching the NCBI non-redundant protein database using blastx (cut-off E-value: 1e-100). Candidate sequences were amplified from larval cDNA by PCR and sequenced (Table S1). CRD regions were predicted by ScanProsite (https://prosite.expasy.org/scanprosite/) (accessed on 10 May 2021). Sequences were aligned with MUSCLE and decorated in Jalview [31]. Phylogenetic trees were constructed in MEGA X with the neighbor-joining method and visualized with Figtree v1.4.4 [32]. Ca^2+^ and sugar-binding sites were predicted by I-TASSER [33]. Molecular graphics were generated using Chimera v1.14 [34]. Sequence logos were generated with WebLogo [35]. Heatmaps were generated using TBtools [36].

### 2.5. Analyses of the Expression Profiles by Real-Time qPCR (RT-qPCR)

To explore the expression profile in different developmental stages, samples from six stages (eggs, early stage larvae, mid-stage larvae, late-stage larvae, pupa, and adults) were ground in liquid nitrogen and stored at −80 °C. To explore the expression profile in naïve larval tissues, fifth instar larvae were anesthetized on ice and dissected to collect hemocytes, fat body, midgut, epidermis, and Malpighian tube. Collected tissues were immediately homogenized in SparkZol reagent (Sparkjade Biotechnology Co., Ltd., Jinan, Shandong, China). For the gene induction analysis, *E. coli*, *S. aureus*, and *B. bassiana* conidia were inactivated with 3% formaldehyde. Moreover, 10^4^ bacteria were resuspended in PBS (10 mM phosphate buffer, 37 mM NaCl, 2.7 mM KCl, pH 7.4) and injected into fifth instar larvae with a microsyringe. PBS was injected as the negative control. *B. bassiana* conidia were resuspended in PBS with 0.05% tween-80, and 4 × 10^4^ conidia were injected into larvae. PBS with 0.05% tween-80 was injected as the negative control. Hemocytes, fat body, and midgut were collected 6 and 24 h post-injection. Three larvae were used in each group, and all experiments were performed in three replicates. cDNA was synthesized from 1 μg total RNA using SPARKscript II RT Plus Kit (with gDNA Eraser). RT-qPCR (95 °C 30 s, 40 cycles of 95 °C 5 s, 60 °C 30 s) was performed using MonAmp™ ChemoHS qPCR Mix (Monad Biotech Co., Ltd., Suzhou, Jiangsu, China) with the CFX96 real-time PCR detection system (Bio-Rad, Hercules, CA, USA). The relative gene expression level was calculated by the 2^−ΔΔCT^ method. Primer information was provided in Table S1.

## 3. Results

### 3.1. Transcriptome Sequencing, Unigene Assembly, and Functional Annotation

A cDNA library was constructed for *M. separata* larvae and sequenced using the Illumina platform. This run produced 47,594,876 raw reads and 44,966,148 clean reads (Clean reads: 94.47%, Q20: 97.45%, Q30: 93.07%). The clean reads were assembled into 81,837 transcripts with a mean length of 1135 bp. Transcripts were further assembled into 45,888 unigenes with a mean length of 910 bp (N50 = 1687 bp). The transcriptome dataset was deposited in Sequence Read Archive (PRJNA702891).

**Figure 1:**
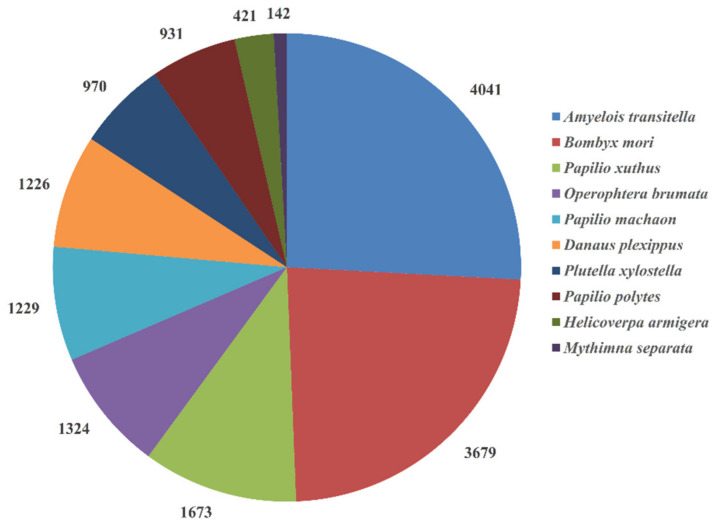
The top 10 species distribution in the NR database. Unigenes were aligned with the NCBI NR protein database. The top 10 species with numbers of matches were shown.

Unigenes were annotated in six databases: NR, GO, KEGG, Pfam, eggNOG, and Swiss-Prot (Table 1). Approximately 18,157 unigenes were annotated to the NR database. The top 10 species with the most matches in the NR database were shown in Figure 1. In total, 4041 unigenes were matched to *Amyelois transitella*, followed by *Bombyx mori* (3679), *Papilio xuthus* (1673), *Operophtera brumata* (1324), *Papilio machaon* (1229), *Danaus plexippus* (1226), *Plutella xylostella* (970), *Papilio polytes* (931), *Helicoverpa armigera* (421), and *Mythimna separata* (142).

**Figure 2:**
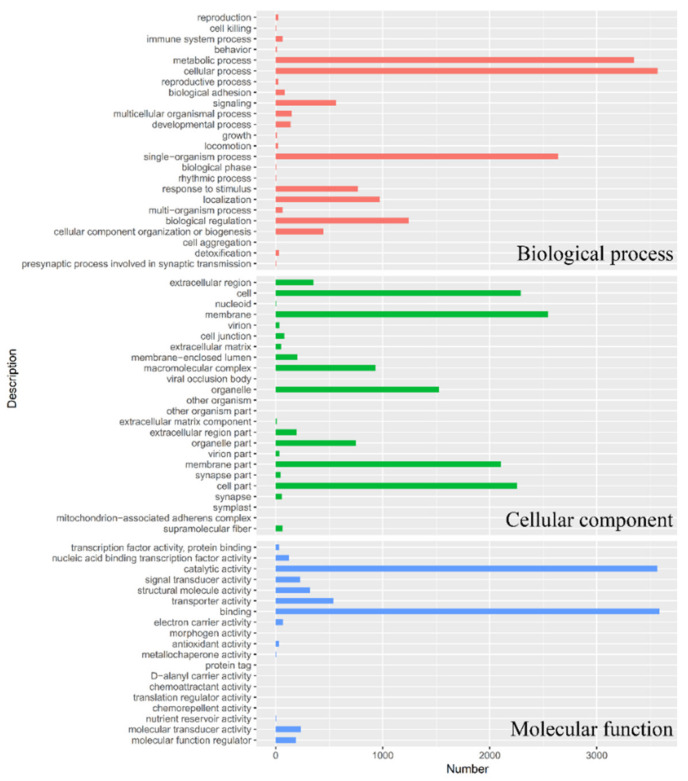
Gene Ontology (GO) classification analysis. Numbers of matched unigenes were plotted against each category for three main categories: biological process, cellular component, and molecular function.

**Figure 3:**
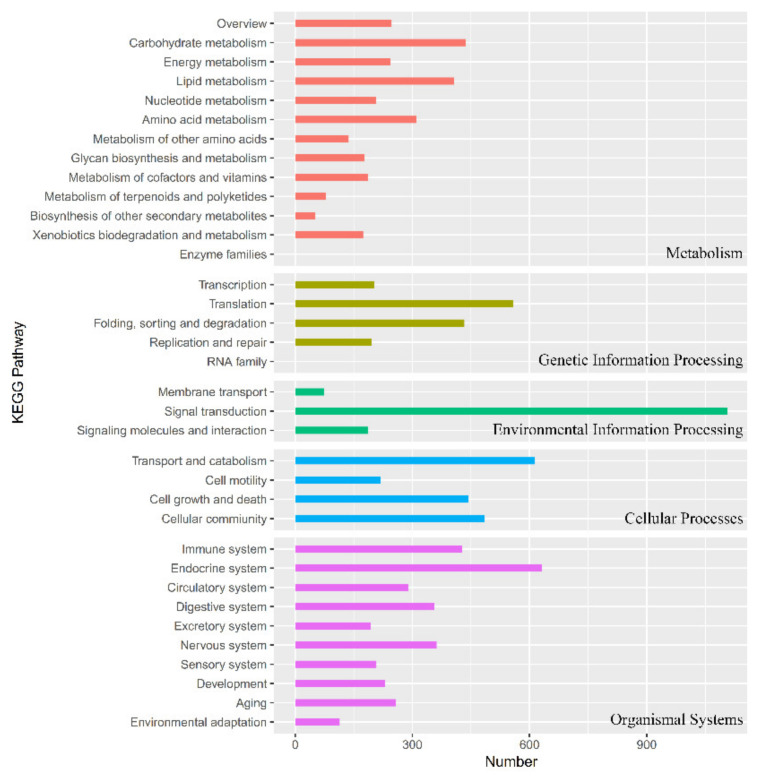
KEGG pathway annotation. KEGG Orthology (KO) and pathway annotations were completed by KEGG Automatic Annotation Server (KAAS) online automated annotation system. Numbers of matched unigenes were plotted against different KEGG pathways.

Functions of unigenes were classified by GO assignments with BLAST2GO (Figure 2). A total of 36,622 unigenes were categorized into 55 categories, which belonged to three main categories: biological process (23), cellular component (19), and molecular function (13). The top 10 categories were binding (3584), cellular process (3569), catalytic activity (3564), metabolic process (3346), single-organism process (2640), membrane (2547), cell (2291), cell part (2257), membrane part (2102), and organelle (1524). For KEGG pathway annotation (Figure 3), the top 10 pathways were signal transduction (1106), endocrine system (632), transport and catabolism (614), translation (559), cellular community (484), cell growth, and death (444), carbohydrate metabolism (436), folding, sorting and degradation (433), immune system (428), and lipid metabolism (406).

### 3.2. Identification of CTLs

**Figure 4:**
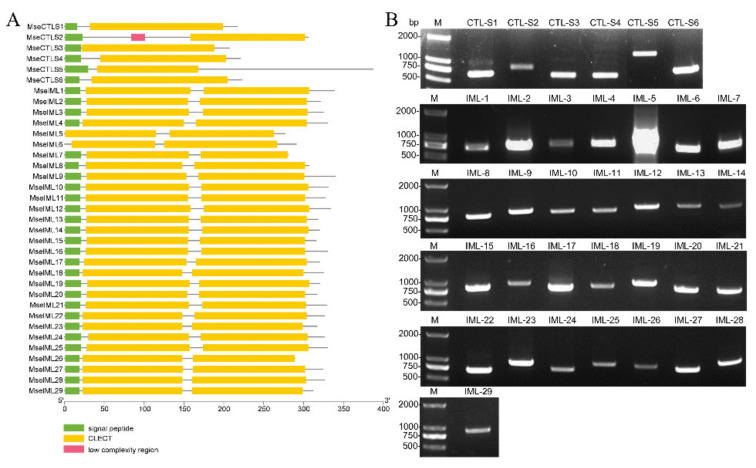
Domain architecture and PCR amplification of *M. separata* CTLs: (**A**) domain architecture of CTLs was analyzed by SMART (http://smart.embl-heidelberg.de/) (accessed on 10 May 2021); and (**B**) CTL genes were amplified using primers in Table S1 and analyzed on 1% agarose gel.

**Figure 5:**
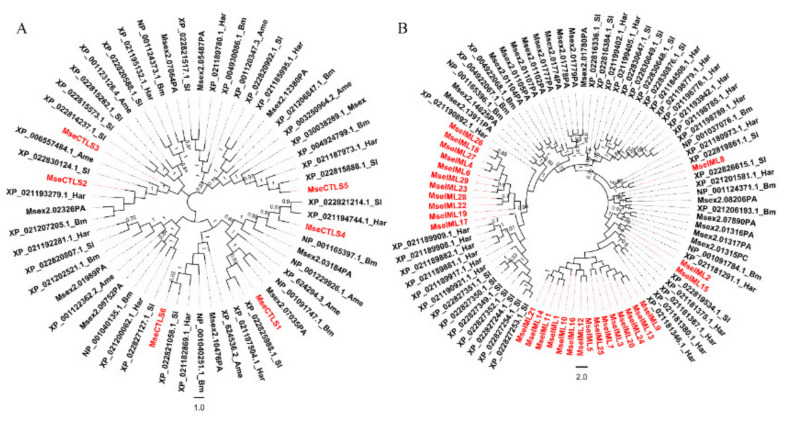
Phylogenetic trees of CTL-S and IML. Protein sequences of 57 CTL-S (**A**) and 90 IML (**B**) were aligned by MUSCLE (https://www.ebi.ac.uk/Tools/msa/muscle/) (accessed on 10 May 2021). The neighbor-joining tree was constructed in MEGA X. The percentage (>70%) of replicate trees in which the associated taxa clustered together in the bootstrap test (1000 replicates) were shown next to the branches as decimals. The evolutionary distances were computed using the p-distance method and are in the units of the number of amino acid differences per site. All ambiguous positions were removed for each sequence pair (pairwise deletion option). *M. separata* CTLs are shown in red. Msex: *Manduca sexta*; Bm: *Bombyx mori*; Ame: *Apis mellifera*; Sl: *Spodoptera litura*; Har: *Helicoverpa armigera*.

Thirty-five unigenes that encode single-CRD or dual-CRD proteins were identified and named CTL-S1–CTL-S6 and IML-1–IML-29 (Figure 4A and Table 2). The coding sequences were amplified from cDNA and correctly sequenced (Figure 4B). To determine the phylogenetic relationships, 57 CTL-S and 90 IML were aligned to construct NJ trees, respectively [37]. All *M. separata* CTL-S clustered with their respective lepidopteran orthologs, except that CTL-S2 and CTL-S3 clustered together (Figure 5A). In contrast, IMLs showed lineage-specific expansions: IML-26/18/27/4/6/29/23/28/22/19 and IML-21/14/11/1/10/16/12/5/25/7/3/20/24/13/9 formed two clusters, indicating that they originated from independent expansions. IML-2 is a 1:1 ortholog of *H. armigera* (XP_021181291.1). IML-8 is a 1:1 ortholog of *S. litura* (XP_022819861.1). IML-17 is clustered with six *H. armigera* and seven *S. litura* orthologs (Figure 5B). These phylogenetic relationships are consistent with previous reports of *B. mori* and *M. sexta* CTLs [7,8].

### 3.3. Sequence and Structure Analyses of CRDs

**Figure 6:**
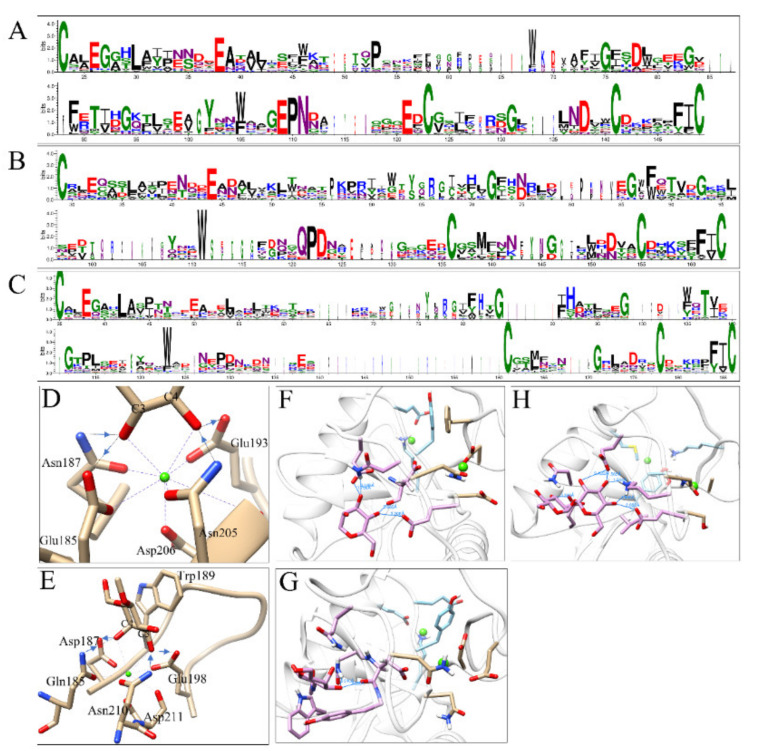
Sequence logos and structures of CRDs. Sequence logos of the ‘EPN-type’ (**A**); ‘QPD-type’ (**B**); and ‘noncanonical-type’ (**C**) CRDs were generated using WebLogo. Amino acids were colored according to chemical properties: polar, green; neutral, purple; basic, blue; acidic, red; hydrophobic, black. The sugar-binding sites of rat MBP-A (PDB: 2MSB) (**D**) and the MBP-A (QPDWG) mutant (PDB: 1AFA) (**E**). The coordination bonds and hydrogen bonds were shown as purple and black dashed lines, respectively. Ca^2+^ ions were shown as green dots. Blue arrows indicate the direction of hydrogen bonds from the hydrogen donors to acceptors. The predicted structures of ‘EPN’ IML-10-CRD1 (**F**), ‘QPD’ IML-14-CRD1 (**G**), and ‘EPD’ IML-17-CRD1 (**H**). Hydrogen bonds were shown as blue lines with distance (Å).

*M. separata* CRDs were analyzed by I-TASSER for ligand type and interacting residues. Of 64 CRDs, 15 contained ‘EPN’ motifs, 12 contained ‘QPD’ motifs, and 37 had noncanonical motifs (Table 3). Sequence logos showed the conserved residues in each type of CRD. Four cysteines were completely conserved in all CRDs. Other conserved residues include: E^26^/G^27^/L^30^/E^38^/P^53^/W^68^/G^75^/D^78^/G^83^/F^89^/T^91^/G^94^/Y^102^/W^105^/E^109^/P^110^/N^111^/E^121^/G^131^/D^138^/F^147^/I^148^ in ‘EPN’-type CRDs (Figure 6A and Figure S1A); E^44^/G^72^/G^86^/F^88^/W^88^/T^90^/G^93^/W^111^/Q^121^/P^122^/D^123^/D^152^/N^152^/F^161^/I^162^ in ‘QPD’-type CRDs (Figure 6B and Figure S1B); E^38^/L^42^/G^84^/H^92^/G^98^/T^107^/W^123^/F^184^/I^185^ in ‘noncanonical’-type CRDs (Figure 6C and Figure S1C). In MBP-A, Glu^185^, Asn^187^, Glu^193^, Asn^205^, and Asp^206^ can form coordination and hydrogen bonds with Ca^2+^ and 3-OH/4-OH, respectively (Figure 6D). The sequence alignment shows that in ‘EPN’-type CRDs, Glu^193^ is completely conserved, Asn^205^ is conserved in 10 out of 15 CRDs, and Asp^206^ is conserved in 14 out of 15 CRDs. In the MBP-A mutant (QPDWG), Gln^185^–Asp^187^–Glu^198^–Asn^210^–Asp^211^ forms a similar coordination and hydrogen bond network. However, the reversed hydrogen donor/acceptor and the adjacent Trp^189^ switched the preferred ligand to galactose (Figure 6E). The sequence comparison showed that Trp^189^ and Asn^210^ were not conserved in insect CRDs. Glu^198^ was conserved in 5 out of 12 CRDs. Asp^211^ was conserved in 8 out of 12 CRDs. In the predicted structures of IML-10-CRD1 (‘EPN’, Figure 6F), IML-14-CRD1 (‘QPD’, Figure 6G), and IML-17-CRD1 (‘EPD’, Figure 6H), sugar ligands were accommodated in proper orientations and positions through hydrogen bonds formed between hydroxyl groups and amino acids.

### 3.4. Spatial and Temporal Expression Profiles

**Figure 7:**
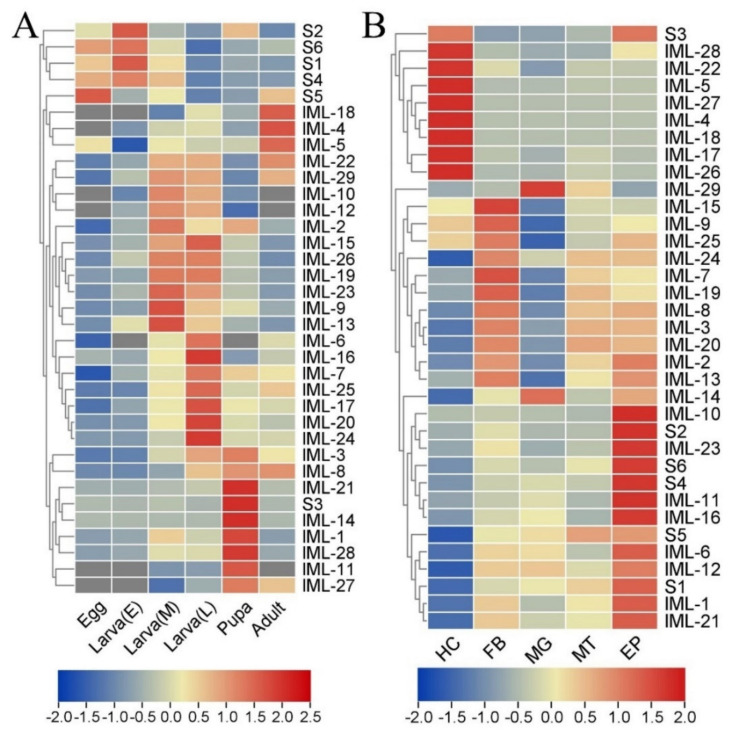
Temporal and spatial expression patterns of *M. separata* CTLs: (**A**) the expression profile of 35 CTLs in eggs, early stage larvae (E), mid-stage larvae (M), late-stage larvae (L), pupa, and adults; (**B**) the expression profile of 35 CTLs in larval tissues. HC, hemocytes; FB, fat body; MG, midgut; MT, Malpighian tube; EP, epidermis. Values (log2) representing the relative expression levels were normalized for each gene (row) and mapped to the color scale on the bottom. Dendrograms were generated by the hierarchical clustering analysis of rows. Genes displaying similar expression patterns were recursively merged into clusters.

To explore the possible functions of CTLs, the expression profile in different developmental stages was analyzed by RT-qPCR. The hierarchical clustering analysis shows distinct expression patterns: *CTL-S1*, *CTL-S2*, *CTL-S4*, *CTL-S5*, and *CTL-S6* mainly express in eggs and early stage larvae; *IML-4*, *IML-5*, and *IML-18* express in adults; *IML-3*, *IML-8*, *IML-21*, *CTL-S3*, *IML-14*, *IML-1*, *IML-28*, *IML-11*, and *IML-27* mainly express in pupa; *IML-6*, *IML-16*, *IML-7*, *IML-25*, *IML-17*, *IML-20*, and *IML-24* express in late-stage larvae; *IML-22*, *IML-29*, *IML-10*, *IML-12*, *IML-2*, *IML-15*, *IML-26*, *IML-19*, *IML-23*, *IML-9*, and *IML-13* express in early and mid-stage larvae (Figure 7A). Larval tissues can produce lots of immune factors to resist the invasion of pathogens. Thus, the expression profile in larval hemocytes, fat body, midgut, Malpighian tube, and epidermis was analyzed. The clustering analysis shows that hemocytes, fat body, and epidermis are the major tissues expressing CTL genes. Notably, *IML-14* and *IML-29* are mostly expressed in the midgut (Figure 7B).

### 3.5. Expression of CTLs Responding to Bacterial Cells and Fungal Spores

**Figure 8:**
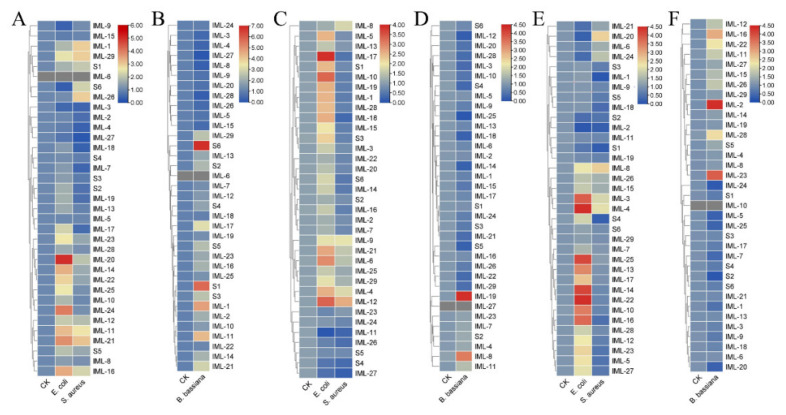
Expression of CTLs responding to *E. coli*, *S. aureus*, and *B. bassiana* conidia after 6 h. Formaldehyde-killed *E. coli*, *S. aureus*, and *B. bassiana* conidia were injected into fifth instar larvae. The expression of CTLs in hemocytes (**A**,**B**), fat body (**C**,**D**), and midgut (**E**,**F**) were analyzed by RT-qPCR. Values (log2) representing the relative expression levels were directly mapped to the color scale without normalization. Dendrograms were generated by the hierarchical clustering analysis of rows.

**Figure 9:**
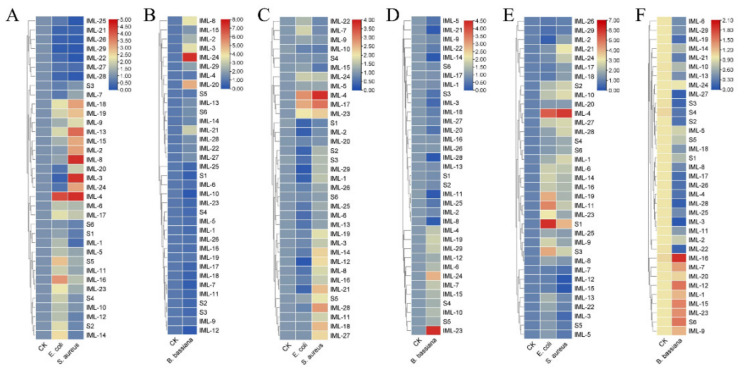
Expression of CTLs responding to *E. coli*, *S. aureus*, and *B. bassiana* conidia after 24 h. Formaldehyde-killed *E. coli*, *S. aureus*, and *B. bassiana* conidia were injected into fifth instar larvae. The expression of CTLs in hemocytes (**A**,**B**), fat body (**C**,**D**), and midgut (**E**,**F**) were analyzed by RT-qPCR. Values (log2) representing the relative expression levels were directly mapped to the color scale without normalization. Dendrograms were generated by the hierarchical clustering analysis of rows.

C-type lectins, especially immulectins, are important for immune responses against bacteria and fungi [17,38]. The expression of CTLs in larval hemocytes, fat body, and midgut responding to *E. coli*, *S. aureus*, and *B. bassiana* were analyzed 6 h and 24 h post-challenge. At 6 hpi, in hemocytes, *IML-20*, *IML-24*, *IML-21*, and *IML-16* were induced by *E. coli*; *IML-21*, *IML-1*, *IML-29*, and *IML-26* were induced by *S. aureus* (Figure 8A); *CTL-S1*, *CTL-S6*, *IML-1*, and *IML-11* were induced by *B. bassiana* (Figure 8B). In the fat body, *IML-17*, *IML-10*, *IML-21*, *IML-6*, *IML-4*, and *IML-12* were induced by *E. coli*; *IML-4*, *IML-8*, *IML-9*, and *IML-12* were induced by *S. aureus* (Figure 8C); *IML-8* and *IML-19* were induced by *B. bassiana* (Figure 8D). In the midgut, *IML-3*, *IML-4*, *IML-25*, *IML-13*, *IML-17*, *IML-14*, *IML-22*, *IML-10*, and *IML-16* were induced by *E. coli*; *IML-20* and *IML-8* were induced by *S. aureus* (Figure 8E); *IML-2*, *IML-16*, and *IML-23* were induced by *B. bassiana* (Figure 8F). At 24 hpi, in hemocytes, IML-4 and IML-16 were induced by *E. coli*; *IML-18*, *IML-19*, *IML-13*, *IML-15*, *IML-2*, *IML-8*, *IML-3*, *IML-24,* and *IML-4* were induced by *S. aureus* (Figure 9A); *IML-24* and *IML-20* were induced by *B. bassiana* (Figure 9B). In the fat body, *IML-4* and *IML-17* were induced by *E. coli*; *IML-4*, *IML-17*, *IML-21*, *IML-28,* and *IML-18* were induced by *S. aureus* (Figure 9C); *IML-23* and *IML-24* were induced by *B. bassiana* (Figure 9D). In the midgut, *IML-4*, *IML-19*, *IML-11*, *CTL-S1,* and *CTL-S3* were induced by *E. coli*; *IML-4* and *CTL-S1* were induced by *S. aureus* (Figure 9E); *IML-16*, *IML-12*, *IML-15*, *IML-23*, and *CTL-S6* were induced by *B. bassiana* (Figure 9F).

## 4. Discussion

With the advancement of the next-generation sequencing (NGS) technique, RNA-seq has become an indispensable tool for studying the transcriptome of non-model organisms, including some agricultural pests [39]. In this study, the transcriptome generated 44,966,148 clean reads and 45,888 unigenes with a mean length of 910 bp, which was comparable to the previous *M. separata* transcriptomes [40,41,42,43,44,45,46,47]. Most homologs of *M. separata* transcripts were found in lepidopteran species, especially in *A. transitella* and *B. mori*. By Gene Ontology (GO) classification and KEGG pathway annotation, unigenes were classified into a variety of biological processes, cellular components, molecular functions, and pathways.

Animal C-type lectin-like domain-containing proteins can be classified into 16 groups based on domain architecture and phylogenetic relationships [48]. In insects, CTLs can be classified based on domain architecture into CTL-S, IML, and CTL-X. CTL-S exist in several insect orders: Lepidoptera, Coleoptera, Hymenoptera, Diptera, and Hemiptera. The numbers of CTL-S vary in different species: *Bombyx mori* (12), *Manduca sexta* (8), *Tribolium castaneum* (10), *Drosophila melanogaster* (30), *Anopheles gambiae* (21), *Aedes aegypti* (37), *Acyrthosiphon pisum* (2), and *Plutella xylostella* (5). Immulectins were almost entirely found in Lepidoptera: *Bombyx mori* (6) and *Manduca sexta* (19) [5]. Here, we identified 6 CTL-S and 29 immulectins from the unigenes of *M. separata* larvae. The phylogenetic analysis showed that CTL-S genes were duplicated in the common ancestor before speciation (Figure 5A), while most IML genes were duplicated after speciation (Figure 5B). Similar phylogenetic relationships were also found in *B. mori* and *M. sexta* [7,8]. Since immulectins broadly participate in regulating the innate immune responses, the expansion of immulectins may greatly improve the survival rates of lepidopteran pests in the natural environment.

The expression profile of developmental stages shows that most IMLs express in larvae and pupae. Only three IMLs (*IML-4*, *IML-5*, and *IML-18*) express in adults. Interestingly, most CTL-S express in eggs and early-stage larvae (Figure 7A). These results suggest that CTL-S may be important for the development of and immunity in embryos and early stage larvae, while IMLs are critical for immunity in larvae, pupae, and adults. A *Periplaneta* lectin participates in the organization or stabilization of the epidermis during leg regeneration [49]. *H. armigera* CTL3 maintains normal larval growth and development by maintaining ecdysone and juvenile hormone signaling and suppressing the abundance of *Enterocuccus mundtii* [50]. CTLs also show a specific spatial expression pattern in naïve larval tissues: hemocytes, fat body, and epidermis are responsible for expressing CTLs (Figure 7B). These are the major larval tissues generating immune molecules. Bacterial and fungal infections induced dramatic changes in the expression of some CTLs (Figure 8 and Figure 9). Our findings are similar to previous transcriptomic studies. In the cotton bollworm *H. armigera*, a transcriptome-based analysis showed that most CTL genes did not undergo any significant changes in the second instar larvae after *B. bassiana* infection, while most of them were upregulated in the fat body of the fifth instar larvae [17]. Although we did not compare the induction of CTLs between early stage and late-stage *M. separata* larvae, we found that most IMLs were expressed in mid-late-stage larvae and pupae. These data suggest that IMLs are important for immune responses in these stages. In the Japanese pine sawyer beetle, *Monochamus alternatus* infected with the entomopathogenic fungus *Metarhizium anisopliae*, several differentially expressed unigenes were CTLs [51]. Twelve CTL genes were identified in *Adelphocoris suturalis* (Hemiptera: Miridae) immune responsive genes against fungal and bacterial pathogens [52]. Fourteen CTLs were identified in immunity-related genes in *Ostrinia furnacalis* against entomopathogenic fungi [53].

A simple rule to predict the ligand specificity of CRDs is based on some key residues: ‘EPN’-motif CRDs can recognize mannose-type ligands; ‘QPD’-motif CRDs usually recognize galactose-type ligands. Mutating ‘EPN’ in MBP-A to ‘QPD’ caused a shift from mannose to galactose ligands [54]. Conversely, mutating ‘QPD’ in sea cucumber CEL-I to ‘EPN’ led to a weak binding affinity for mannose [55]. Some surrounding residues or structures also can affect ligand selection. An additional mutation of Trp^105^ to His in MBP-A further increased the affinity to mannose [55]. A glycine-rich loop helps to exclude mannose and accommodate galactose in Gal-type CTLs [56,57]. However, there are exceptions to this rule. CEL-IV, a CTL in sea cucumber, *Cucumaria echinate*, contains the ‘EPN’ motif but binds galactose [58]. TC14, a CTL from the tunicate *Polyandrocarpa misakiensis* contains ‘EPS’ but binds galactose [59]. Some CRDs bind carbohydrates in the absence of the ‘EPN/QPD’ motif or Ca^2+^. The CRD of eosinophil major basic protein (EMBP) binds to heparin and heparan sulfate at a different contact site through electrostatic interactions and hydrogen bonds [60]. Bivalve lectins SPL-1 and SPL-2, which contained ‘RPD’ and ‘KPD’ motifs, showed Ca^2+^-independent binding affinity for GlcNAc or GalNAc [61]. Structural studies have elucidated how these residues interact with Ca^2+^ and carbohydrates. For monosaccharide ligands, steric restrictions are imposed by the coordination bonds and hydrogen bonds formed between 3-OH/4-OH, Ca^2+^, and ‘EPN/QPD’ motifs. Mannose has equatorial 3-OH and equatorial 4-OH, while galactose has equatorial 3-OH and axial 4-OH. In addition, the hydrogen donors and acceptors are reversed between E(acceptor)-P-N(donor) and Q(donor)-P-D(acceptor) (Figure 6D,E). Therefore, the predicted ligand specificity needs to be verified experimentally.

To sum up, this study built a de novo transcriptome assembly of *M. separata* larvae, from which 6 ‘S-type’ and 29 ‘IML-type’ CTLs were identified. Sequence features, phylogenetic relationships, ligand specificity, and expression profiles were studied. Further studies are required to explore the function of each CTL in the oriental armyworm.

## Figures and Tables

**Table 1:** Functional annotation of unigenes in databases.

Database	Number	Percentage
NR	18,157	39.57
GO	8035	17.51
KEGG	8836	19.26
Pfam	9527	20.76
eggNOG	17,125	37.32
Swiss-Prot	11,142	24.28
In all database	3564	7.77

**Table 2:** *M. separata CTL* genes identified from unigenes.

Name	Accession Number	ORF (aa)	BLASTX Best Hit			
			Reference Organism	Accession Number	E Value	Identity (%)
CTL-S1	MW658722	217	*Manduca sexta*	XP_030027697.2	4 × 10^−147^	99
CTL-S2	MW658723	306	*Spodoptera litura*	XP_022830124.1	2 × 10^−179^	86
CTL-S3	MW658724	207	*Helicoverpa armigera*	XP_021189783.1	5 × 10^−143^	100
CTL-S4	MW658725	221	*Manduca sexta*	XP_030021432.1	1 × 10^−149^	95
CTL-S5	MW658726	387	*Spodoptera litura*	XP_022815888.1	0	89
CTL-S6	MW658727	223	*Spodoptera litura*	XP_022821058.1	3 × 10^−152^	99
IML-1	MW658728	339	*Spodoptera frugiperda*	XP_035436562.1	6 × 10^−131^	58
IML-2	MW658729	321	*Spodoptera frugiperda*	XP_035436562.1	0	77
IML-3	MW658730	325	*Spodoptera exigua*	KAF9421847.1	6 × 10^−122^	54
IML-4	MW658731	330	*Mythimna separata*	BBC20960.1	2 × 10^−165^	71
IML-5	MW658732	277	*Spodoptera exigua*	AQX37248.1	6 × 10^−106^	54
IML-6	MW658733	291	*Mythimna separata*	BBC20960.1	3 × 10^−118^	62
IML-7	MW658734	281	*Spodoptera frugiperda*	XP_035436562.1	2 × 10^−104^	53
IML-8	MW658735	307	*Trichoplusia ni*	XP_026724737.1	9 × 10^−150^	65
IML-9	MW658736	340	*Spodoptera frugiperda*	XP_035436562.1	3 × 10^−110^	51
IML-10	MW658737	331	*Spodoptera exigua*	AQX37248.1	2 × 10^−110^	51
IML-11	MW658738	327	*Spodoptera frugiperda*	XP_035436562.1	1 × 10^−112^	50
IML-12	MW658739	334	*Spodoptera frugiperda*	XP_035436562.1	8 × 10^−111^	54
IML-13	MW658740	318	*Spodoptera frugiperda*	XP_035436562.1	4 × 10^−114^	54
IML-14	MW658741	320	*Spodoptera frugiperda*	XP_035436562.1	2 × 10^−121^	57
IML-15	MW658742	316	*Spodoptera frugiperda*	XP_035436562.1	3 × 10^−119^	56
IML-16	MW658743	330	*Spodoptera frugiperda*	XP_035436562.1	3 × 10^−116^	52
IML-17	MW658744	320	*Mythimna separata*	BAV01248.1	0	98
IML-18	MW658745	325	*Mythimna separata*	BBC20960.1	1 × 10^−146^	63
IML-19	MW658746	320	*Mythimna separata*	BAV01248.1	9 × 10^−127^	59
IML-20	MW658747	317	*Spodoptera frugiperda*	XP_035436562.1	4 × 10^−121^	56
IML-21	MW658748	329	*Spodoptera frugiperda*	XP_035436562.1	1 × 10^−120^	54
IML-22	MW658749	326	*Trichoplusia ni*	XP_026732761.1	1 × 10^−112^	53
IML-23	MW658750	317	*Mythimna separata*	BAV01248.1	2 × 10^−120^	57
IML-24	MW658751	326	*Spodoptera frugiperda*	XP_035436562.1	8 × 10^−126^	55
IML-25	MW658752	330	*Spodoptera frugiperda*	XP_035436562.1	1 × 10^−119^	59
IML-26	MW658753	289	*Mythimna separata*	BBC20960.1	3 × 10^−12^	62
IML-27	MW658754	324	*Mythimna separata*	BBC20960.1	0	99
IML-28	MW658755	326	*Mythimna separata*	BBC20960.1	2 × 10^−135^	59
IML-29	MW658756	312	*Trichoplusia ni*	XP_026732761.1	1 × 10^−142^	64

**Table 3:** Sequence features of CRDs.

CRD	Motif	PDB Hit	Ca^2+^ Coordinators ^2^	Ligand ^1^	Ligand Binding Residues ^2^	Disulfide Bridges
CTL-S1	QPD	1dv8A	38,40,44,158 [0.14]	GQ2	59,115,117,118,132,145,146,147,153 [0.48]	70–198; 171–190
CTL-S2	EPN	4kzvA	62,66,90,99,100 [0.39]	MAN	87,89,99,115,116,118 [0.87]	197–301; 275–293
CTL-S3	VPQ	1htnA	36,38,42,160 [0.13]	1SL5A	104,105,106,107,108,109,110,111,116,117,119,120,121,122,123,124,125,126,148,153 [0.24]	55–187; 164–179
CTL-S4	QPD	4kzvA	35,37,41,154 [0.13]	4RS	56,112,114,124,130,141,142,147 [0.51]	75–202; 175–194
CTL-S5	-	1wmyA	31,33,37,121 [0.12]	GQ2	53,85,87,88,98,108,109,110,116 [0.35]	69–167; 143–159
CTL-S6	QPD	4kzvA	38,40,44,163 [0.10]	BM3	120,122,124,133,150,151,152 [0.50]	71–204; 177–196
IML-1-CRD1	QPD	4kzvA	56,60,85,90,91 [0.44]	NGA	49,82,84,86,90,94,96,105,106 [0.57]	57–157; 132–149
IML-1-CRD2	QPD	4yliA	31,33,37,126 [0.22]	291	93,95,101,107,113,114,115 [0.32]	203–306; 284–298
IML-2-CRD1	EPN	4yliA	55,59,62,84,89,90 [0.27]	MAN	81,83,89,101,102,104 [0.73]	58–154; 132–146
IML-2-CRD2	QPD	1wmyA	31,33,37,126 [0.13]	4RS	61,94,96,101,107,113,114,119 [0.66]	200–303; 281–295
IML-3-CRD1	QPD	4yliA	56,60,85,90,91 [0.38]	GQ2	49,82,84,86,94,102,103,104,110 [0.58]	58–155; 133–147
IML-3-CRD2	QPE	4yliA	31,33,37,125 [0.16]	4RS	61,93,95,100,106,112,113,118 [0.51]	202–304; 282–296
IML-4-CRD1	EPD	4yliA	24,26,30,114 [0.03]	MAN	81,83,89,101,102,104 [0.81]	51–149; 127–141
IML-4-CRD2	EPN	4yliA	31,33,37,131 [0.11]	4RS	64,98,100,106,112,118,119,124 [0.64]	195–303; 281–295
IML-5-CRD1	QPD	4yliA	56,60,63,85,90,91 [0.10]	TRE	49,82,84,86,90,96,102,103,108 [0.67]	17–114; 92–106
IML-5-CRD2	KPA	4yliA	29,31,35,123 [0.23]	BM3	92,94,96,98,110,111,112 [0.21]	160–262; 240–254
IML-6-CRD1	EPD	4yliA	37,41,44,66,71,72 [0.15]	NAG	63,65,71,83,84,89 [0.79]	19–114; 91–105
IML-6-CRD2	EPN	4yliA	31,33,37,131 [0.11]	MAN	61,98,100,106,118,119,121 [0.67]	158–266; 244–258
IML-7-CRD1	QPS	4yliA	61,65,90,95,96 [0.38]	MAN	87,89,95,107,108,110 [0.65]	58–155; 133–147
IML-7-CRD2	LPQ	4yliA	66,70,72,95,98,99 [0.15]	MAN	92,94,96,98 [0.39]	201-?; 281-?
IML-8-CRD1	NTD	1htnA	40,42,46,122 [0.16]	NGA	63,89,90,91,92,109,110,111 [0.23]	57–147; 125–139
IML-8-CRD2	EPN	1jznA	27,29,33,127 [0.10]	MAN	54,93,95,101,114,115,117 [0.72]	193–301; 278–293
IML-9-CRD1	HPD	4yliA	31,33,37,117 [0.16]	4RS	54,87,89,92,98,104,105,110 [0.45]	58–152; 130–144
IML-9-CRD2	QAD	4yliA	31,33,37,125 [0.22]	GQ2	61,92,94,95,104,112,113,114,120 [0.34]	198–300; 278–292
IML-10-CRD1	EPN	4yliA	56,60,85,90,91 [0.22]	MAN	82,84,90,103,104,106 [0.71]	57–155; 132–147
IML-10-CRD2	QPD	4yliA	29,31,35,125 [0.20]	NGA	59,92,94,96,100,104,106,112,113 [0.20]	201–305; 283–297
IML-11-CRD1	TPD	4yliA	56,60,85,90,91 [0.24]	MAN	82,84,90,102,103,105 [0.62]	57–154; 132–146
IML-11-CRD2	KPD	4yliA	31,33,37,124 [0.14]	GAL	94,96,99,111,112 [0.21]	200–301; 279–293
IML-12-CRD1	TPD	4kzvA	56,60,85,90,91 [0.27]	NGA	49,82,84,86,90,94,96,105,106 [0.55]	57–157; 132–149
IML-12-CRD2	EPD	4yliA	31,33,37,126 [0.19]	GQ2	61,93,95,96,105,113,114,115,121 [0.52]	203–306; 284–298
IML-13-CRD1	EPN	4yliA	57,61,86,90,91 [0.23]	MAN	83,85,90,102,103,105 [0.69]	59–155; 133–147
IML-13-CRD2	QPD	4yliA	31,33,37,125 [0.21]	4RS	61,93,95,100,106,112,113,118 [0.47]	201–303; 281–295
IML-14-CRD1	QPD	4yliA	56,60,63,86,91,92 [0.35]	MAN	83,85,91,103,104,106 [0.67]	57–155; 133–147
IML-14-CRD2	QPD	4yliA	31,33,37,127 [0.32]	GQ2	61,94,96,97,106,114,115,116,122 [0.09]	201–305; 283–297
IML-15-CRD1	EPN	4yliA	56,60,63,85,89,90 [0.29]	MAN	82,84,89,101,102,104 [0.70]	58–154; 132–146
IML-15-CRD2	SRL	4yliA	31,33,37,124 [0.24]	GQ2	61,91,93,94,103,111,112,113,119 [0.30]	200–301; 279–293
IML-16-CRD1	EPN	4yliA	56,60,85,90,91 [0.23]	MAN	82,84,90,102,103,105 [0.70]	57–154; 132–146
IML-16-CRD2	QPV	4yliA	31,33,37,124 [0.13]	MMA	94,96,98,99,111,112,113 [0.18]	200–301; 279–293
IML-17-CRD1	EPD	4yliA	18,20,24,109 [0.05]	MAN	76,78,84,96,97,99 [0.79]	53–152; 130–144
IML-17-CRD2	EPN	4yliA	31,33,37,128 [0.11]	TRE	61,95,97,99,103,109,115,116,121 [0.63]	198–303; 281–295
IML-18-CRD1	EPD	4yliA	18,20,24,106 [0.04]	MAN	73,75,81,93,94,96 [0.78]	51–147; 125–139
IML-18-CRD2	EPT	4yliA	-	4RS	63,97,99,104,110,116,117,122 [0.68]	193–299, 277–291
IML-19-CRD1	EPD	4yliA	26,28,32,117 [0.04]	TRE	51,84,86,88,92,98,104,105,110 [0.79]	57–156; 134–148
IML-19-CRD2	NPD	1b08A	31,33,37,127 [0.18]	GQ2	61,95,97,98,106,114,115,116,122 [0.56]	202–306; 284–298
IML-20-CRD1	QPN	4yliA	26,28,32,113 [0.07]	MAN	82,84,89,100,101,103 [0.66]	58–153; 132–145
IML-20-CRD2	QPI	4yliA	31,33,37,126 [0.15]	NGA	94,96,98,101,113,114,115 [0.41]	198–301; 279–293
IML-21-CRD1	IPD	4yliA	56,60,86,91,92 [0.23]	TRE	49,83,85,87,91,97,103,104,109 [0.62]	57–155; 133–147
IML-21-CRD2	QPV	1wmyA	31,33,37,124 [0.21]	NAG	91,93,100,111,112,113 [0.35]	201–302; 280–294
IML-22-CRD1	EPD	4yliA	26,28,32,114 [0.04]	TRE	48,81,83,85,89,95,101,102,107 [0.81]	51–147; 125–139
IML-22-CRD2	KPT	4yliA	31,33,37,133 [0.17]	GQ2	67,101,103,104,112,120,121,122,128 [0.54]	193–303; 281–295
IML-23-CRD1	EPD	4yliA	26,28,32,114 [0.04]	MAN	81,83,89,101,102,104 [0.81]	51–147; 125–139
IML-23-CRD2	DPN	4yliA	31,33,37,129 [0.10]	4RS	61,95,97,104,110,116,117,122 [0.64]	192–298; 276–290
IML-24-CRD1	EPN	4yliA	56,60,85,90,91 [0.36]	MAN	82,84,90,102,103,105 [0.68]	58–155; 133–147
IML-24-CRD2	VPT	4yliA	31,33,37,126 [0.13]	GQ2	61,94,96,97,105,113,114,115,121 [0.27]	201–304; 282–296
IML-25-CRD1	EPN	4yliA	57,61,64,86,91,92 [0.16]	MAN	83,85,91,103,104,106 [0.69]	58–156; 134–148
IML-25-CRD2	QPG	4yliA	29,31,35,124 [0.19]	GQ2	59,92,94,95,103,111,112,113,119 [0.31]	202–305; 283–297
IML-26-CRD1	EPD	4kzvA	34,36,40,122 [0.04]	NGA	56,89,91,93,97,101,103,109,110 [0.78]	51–147; 125–139
IML-26-CRD2	EPT	3zhgA	-	MMA	97,99,101,104 [0.23]	194-?; 278-?
IML-27-CRD1	EPD	4yliA	24,26,30,113 [0.04]	MAN	80,82,88,100,101,103 [0.80]	51–148; 126–140
IML-27-CRD2	EPN	4yliA	31,33,37,130 [0.14]	FUC	98,100,102,105,117,118,119 [0.32]	194–301; 279–293
IML-28-CRD1	EPD	4yliA	24,26,30,112 [0.04]	MAN	79,81,87,99,100,102 [0.81]	51–147; 125–139
IML-28-CRD2	EPN	4yliA	31,33,37,133 [0.14]	4RS	67,101,103,108,114,120,121,126 [0.59]	192–302; 280–294
IML-29-CRD1	EPD	4yliA	26,28,32,114 [0.04]	NGA	48,81,83,85,89,93,95,101,102 [0.80]	51–147; 125–139
IML-29-CRD2	EPN	4yliA	68,72,75,98,103,104 [0.03]	TRE	61,95,97,99,103,109,115,116,121 [0.65]	193–298; 276–290

^1^ GQ2: 6-o-alpha-d-glucopyranosyl-4-o-sulfo-alpha-d-glucopyranose; MAN: alpha-d-mannose; 1SL5A: alpha-l-fucopyranose-(1-3)-[beta-d-galactopyranose-(1-4)]2-acetamido-2-deoxy-beta-d-glucopyranose-(1-3)-beta-d-galactopyranose; 4RS: 6-butanoyl-trehalose; BM3: n-acetyl-alpha-d-mannosamine; NGA: n-acetyl-d-galactosamine; 291: prop-2-en-1-yl 7-o-carbamoyl-l-glycero-alpha-d-manno-heptopyranoside; TRE: trehalose; NAG: aldehydo-n-acetyl-d-glucosamine; GAL: beta-d-galactose; MMA: methyl alpha-d-mannopyranoside; FUC: alpha-l-fucopyranose. ^2^ C-scores of predicted Ca^2+^ and sugar ligands are shown in []. C-score is the confidence score of the prediction [0–1], where a higher score indicates a more reliable prediction.

## Data Availability

Not applicable.

## Supplementary Materials

The following are available online at https://www.mdpi.com/article/10.3390/insects12060559/s1, Figure S1: Sequence alignment of three types of CRDs; Table S1: List of Primers.

## Abbreviations

PRR: pattern recognition receptor; PAMP: pathogen-associated molecular pattern; CTL: C-type lectin; PGRP: peptidoglycan-recognition protein; βGRP: β-1,3-glucan recognition protein; CRD: carbohydrate-recognition domain; CTLD: C-type lectin-like domain; IML: immulectin; PBS, phosphate-buffered saline; RT-qPCR: quantitative reverse transcription PCR; SMART: Simple Modular Architecture Research Tool; I-TASSER: Iterative Threading Assembly Refinement.
