# Supplementary material for: Identification of 35 C-Type Lectins in the Oriental Armyworm, Mythimna separata (Walker)
Source: Insects. 2021 Jun 16;12(6):559. doi: 10.3390/insects12060559 (PMC8235521; doi:10.3390/insects12060559)
Supplement: Supplementary file 1 [file insects-12-00559-s001.zip › Figure S1. CRD alignment.pdf]

A

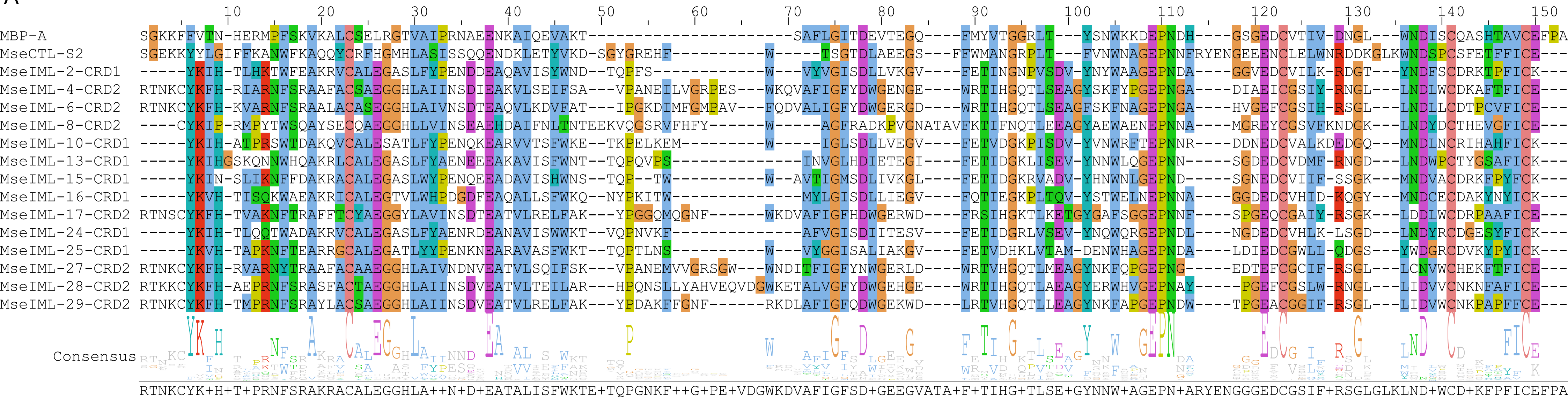
10 20 30 40 50 60 70 80 90 100 110 120 130 140 150
MBP-A SGKKFFVIN-HERMPFSKVKALCSELRGTVAIPRNAEENKAIQEVAKT----------------------SAFLGITDEVTEGQ----FMYVTGGRLT---YSNWKKDEPNDH----GSGEDCVTIV-DNGL---WNDISCQASHTAVCEFPA
MseCTL-S2 SGEKKYYLGIFFKANWFKAQQYCRFHGMHLASISSQQENDKLETYVKD-SGYGREHF----------W-----TSGTDLAEEGS---FFWMANGRPLT---FVNWNAGEPNNFRYENGEEENCLELWNRDDKGLKWNDSPCSFETFFICE---
MseIML-2-CRD1 -----YKIH-TLHKTWFEAKRVCALEGASLFYPENDDEAQAVISYWND--TQPFS-----------W---VYVGISDLLVKGV----FETINGNPVSDV-YNYWAAGEPNDA----GGVEDCVILK-RDGT---YNDFSCDRKTPFICK---
MseIML-4-CRD2 RTNKCYKFH-RIARNFSRAAFACSAEGGHLAIINSDIEAKVLSEIFSA---VPANEILVGRPES---WKQVAFIGFYDWGENGE----WRTIHGQTLSEAGYSKFYPGEPNGA----DIAEICGSIY-RNGL---LNDLWCDKAFTFICE---
MseIML-6-CRD2 RTNKCYKFH-KVARNFSRAALACSAEGGHLAIVNSDTEAQVLKDVFAT---IPGKDIMFGMPAV---FQDVALIGFYDWGERGD----WRTIHGQTLSEAGFSKFNAGEPNGA----HVGEFCGSIH-RSGL---LNDLLCDTPCVFICE---
MseIML-8-CRD2 ----CYKIP-RMPYTWSQAYSECQAEGGHLLVINSEAEHDAIFNLTNTEEKVQGSRVFHFY------W------AGFRADKPVGNATAVFKTIFNQTLEEAGYAEWAENEPNNA----MGREYCGSVFKNDGK---LNDYDCTHEVGFICE---
MseIML-10-CRD1 -----YKIH-ATPRSWTDAKQVCALESATLFYPENQKEARVVTSFWKE--TKPELKEM---------W------IGLSDLLVEGV----FETVDGKPISDV-YVNWRFTEPNNR----DDNEDCVALKDEDGQ---MNDLNCRIHAHFICK---
MseIML-13-CRD1 -----YKIHGSKQNNWHQAKRLCALEGASLFYAENEEEAKAVISFWNT--TQPQVPS---------------INVGLHDIETEGI----FETIDGKLISEV-YNNWLQGEPNN-----SGDEDCVDMF-RNGD---LNDWPCTYGSAFICK---
MseIML-15-CRD1 -----YKIN-SLIKNFFDAKRACALEGASLWYPENQEEADAVISHWNS--TQP--TW----------W--AVTIGMSDLIVKGL----FETIDGKRVADV-YHNWNLGEPND-----SGNEDCVIIF-SSGK---MNDVACDRKFPYFCK---
MseIML-16-CRD1 -----YKVH-TISQKWAEAKRICALEGTVLWHPDGDFEAQALLSFWKQ--NYPKITW---------------MYLGISDLLIEGV----FQTIEGKPLTQV-YSTWELNEPNNA----GGDEDCVHID-KQGY---MNDCECDAKYNYICK---
MseIML-17-CRD2 RTNSCYKFH-TVAKNFTRAFFTCYAEGGYLAVINSDTEATVLRELFAK---YPGGQMQGNF------WKDVAFIGFHDWGERWD----FRSIHGKTLKETGYGAFSGGEPNNF----SPGEQCGAIY-RSGK---LDDLWCDRPAAFICE---
MseIML-24-CRD1 -----YKIH-TLQQTWADAKRVCALEGASLFYAENRDEANAVISWWKT--VQPNVKF---------------AFVGISDIITESV----FETIDGRLVSEV-YNQWQRGEPNDL----NGDEDCVHLK-LSGD---LNDYRCDGESYFICK---
MseIML-25-CRD1 -----YKVH-TAPKNFTEARRGCALEGATLYYPENKNEARAVASFWKT--TQPTLNS----------W---VYGGISALIAKGV----FETVDHKLVTAM-DENWHAGEPNDA----LDIEDCGWLL-QDGS---YWDGRCDVKYPYICK---
MseIML-27-CRD2 RTNKCYKFH-RVARNYTRAAFACAAEGGHLAIVNDNVEATVLSQIFSK---VPANEMVVGRSGW---WNDITFIGFYNWGERLD----WRTVHGQTLMEAGYNKFQPGEPNG-----EDTEFCGCIF-RSGL---LCNVWCHEKFTFICE---
MseIML-28-CRD2 RTKKCYKFH-AEPRNFSRASFACTAEGGHLAIINSDVEATVLTEILAR---HPQNSLLYAHVEQVDGWKETALVGFYDWGEHGE----WRTIHGQTLAEAGYERWHVGEPNAY-----PGEFCGSLW-RNGL---LIDVVCNKNFAFICE---
MseIML-29-CRD2 RTNKCYKFH-TMPRNFSRAYLACSAEGGHLAIINSDVEATVLRELFAK---YPDAKFFGNF------RKDLAFIGFQDWGEKWD----LRTVHGQTLLEAGYNKFAPGEPNDW----TPGEACGGIF-RSGL---LIDVWCNKPAPFFCE---
Consensus
RTNKCYK+H+T+PRNFSRAKRACALEGGHLA++N+D+EATALISFWKTE+TQPGNKF++G+PE+VDGWKDVAFIGFSD+GEEGVATA+F+TIHG+TLSE+GYNNW+AGEPN+ARYENGGGEDCGSIF+RSGLGLKLND+WCD+KFPFICEFPA

B

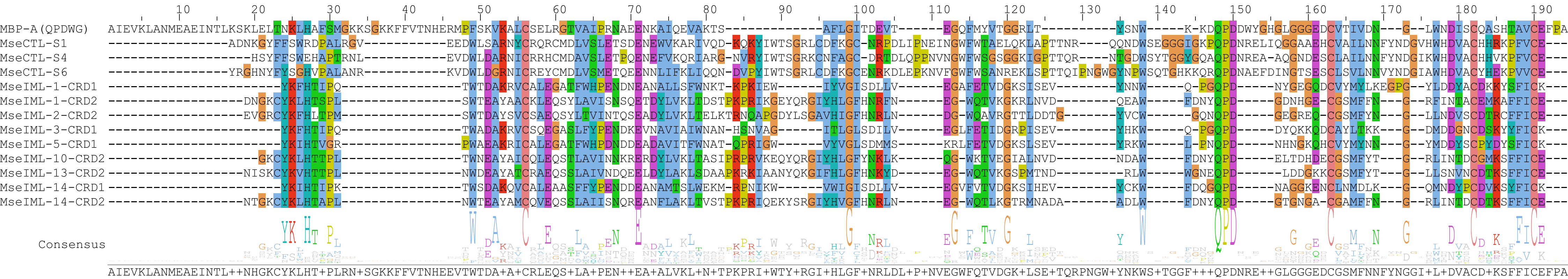
10 20 30 40 50 60 70 80 90 100 110 120 130 140 150 160 170 180 190
MBP-A(QPDWG) AIEVKLANMEAEINTLKSKLELTNKLHAFSMGKKSGKKFFVTNHERMPFSKVKALCSELRGTVAIPRNAEENKAIQEVAKTS------------AFLGITDEVT-------EGQFMYVTGGRLT----------YSNW-----K-KDQPDDWYGHGLGGGEDCVTIVDN---G--LWNDISCQASHTAVCEFPA
MseCTL-S1 ----------------ADNKGYFFSWRDPALRGV-----------EEDWLSARNYCRQRCMDLVSLETSDENEWVKARIVQD-KQKYIWTSGRLCDFKGC-NRPDLIPNEINGWFWTAELQKLAPTTNR----QQNDWSEGGGIGKPQPDNRELIQGGAAEHCVAILNNFYNDGVHWHDVACHHRKPFVCE---
MseCTL-S4 -------------------HSYFFSWEHAPTRNL-----------EVDWLDARNICRRHCMDLVSLETPQENEFVKQRIARG-NVRYIWTSGRKCNFAGC-DRTDLQPPNVNGWFWSGSGGKIGPTTQR----NTGDWSYTGGYGQAQPDNREA-AQGNDESCLAILNNFYNDGIKWHDVACHHVKPFVCE---
MseCTL-S6 ----------------YRGHNYFYSGHVPALANR-----------KVDWLDGRNICREYCMDLVSMETQEENNLIFKLIQQN-DVPYIWTSGRLCDFKGCENRKDLEPKNVFGWFWSANREKLSPTTQIPNGWGYNPWSQTGHKKQRQPDNAEFDINGTSESCLSVLNNVYNDGIAWHDVACYHEKPVVCE---
MseIML-1-CRD1 ----------------------YKFHTIPQ------------------TWTDAKRVCALEGATFWHPENDNEANALLSFWNKT-KPKIEW-------IYVGISDLLV-------EGAFETVDGKSISEV----------YNNW-----Q-PGQPD-----NYGEGQDCVYMYLKEGPG--YLDDYACDKKYSFICK---
MseIML-1-CRD2 ------------------DNGKCYKLHTSPL------------------SWTEAYAACKLEQSYLAVISNSQETDYLVKLTDSTPKPRIKGEYQRGIYHLGFHNRFN-------EG-WQTVKGKRLNVD----------QEAW----FDNYQPD-----GDNHGE-CGSMFFN---G--RFINTACEMKAFFICE---
MseIML-2-CRD2 ------------------EVGRCYKFHLTPM------------------SWTDAYSVCSAEQSYLTVINTQSEADYLVKLTELKTRNQAPGDYLSGAVHIGFHNRLN-------DG-WQAVRGTTLDDTG----------YVCW-----GQNQPD-----GEGREQ-CGSMFYN---G--LLNDVSCDTRCFFICE---
MseIML-3-CRD1 ----------------------YKFHTIPQ------------------TWADAKRVCSQEGASLFYPENDKEVNAVIAIWNAN-HSNVAG-------ITLGLSDILV-------EGLFETIDGRPISEV----------YHKW-----Q-PGQPD-----DYQKKQDCAYLTKK---G--DMDDGNCDSKYYFICK---
MseIML-5-CRD1 ----------------------YKIHTVGR------------------PWAEAKRICALEGATFWHPDNDDEADAVITFWNAT-QPRIGW-------VYVGLSDMMS-------KRLFETVDGKSLSEV----------YRKW-----L-PNQPD-----NHNGKQHCVYMYNN---G--YMDDYSCPYDYSFICK---
MseIML-10-CRD2 --------------------GKCYKLHTTPL------------------TWNEAYAICQLEQSTLAVINNKRERDYLVKLTASIPRPRVKEQYQRGIYHLGFYNKLK-------QG-WKTVEGIALNVD----------NDAW----FDNYQPD-----ELTDHDECGSMFYT---G--RLINTDCGMKSFFICE---
MseIML-13-CRD2 ------------------NISKCYKVHTTPL------------------NWDEAYATCRAEQSSLAIVNDQEELDYLAKLSDAAPKRKIAANYQKGIFHLGFHNKYD-------EG-WQTVKGSPMTND----------RLW----WGNEQPD-----LDDGKKCGSMFYT---G--LLSNVNCDTKSFFICE---
MseIML-14-CRD1 ----------------------YKIHTIPK------------------TWSDAKQVCALEAASFFYPENDDEANAMTSLWEKM-RPNIKW-------VWIGISDLLV-------EGVFVTVDGKSIHEV----------YCKW-----FDQGQPD-----NAGGKENCLNMDLK---G--QMNDYPCDVKSYFICK---
MseIML-14-CRD2 ------------------NTGKCYKLHTAPL------------------NWTEAYAMCQVEQSSLAIISNQREANFLAKLTVSTPKPRIQEKYSRGIYHVGFHNRLN-------EG-WQTLKGTRMNADA----------ADLW----FDNYQPD-----GTGNGA-CGAMFFN---G--RLINTDCDTKSFFICE---
Consensus
AIEVKLANMEAEINTL++NHGKCYKLHT+PLRN+SGKKFFVTNHEEVTWTDA+A+CRLEQS+LA+PEN++EA+ALVKL+N+TPKPRI+WTY+RGI+HLGF+NRLDL+P+NVEGWFQTVDGK+LSE+TQRPNGW+YNKWS+TGGF+++QPDNRE++GLGGGEDCGSMFNNFYNGGI+L+DVACD+KSFFICEFPA

C

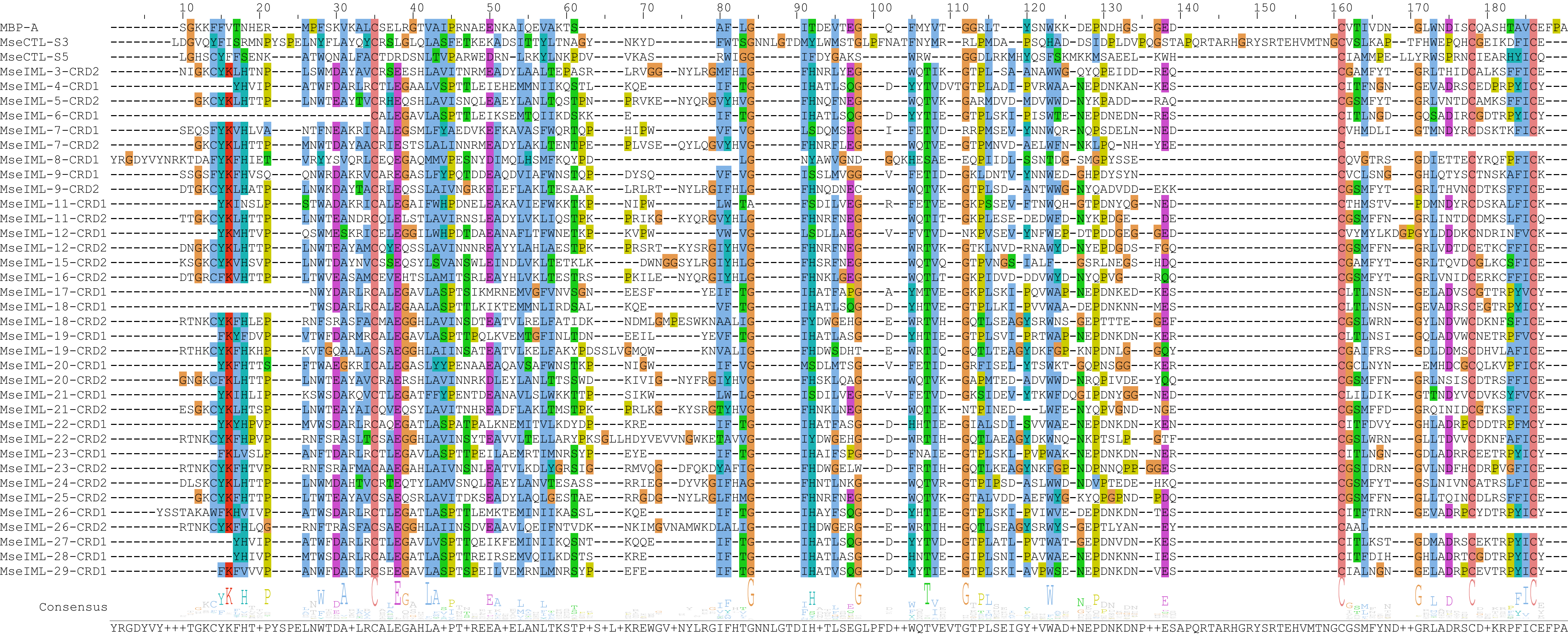
MBP-A
MseCTL-S3
MseCTL-S5
MseIML-3-CRD2
MseIML-4-CRD1
MseIML-5-CRD2
MseIML-6-CRD1
MseIML-7-CRD1
MseIML-7-CRD2
MseIML-8-CRD1
MseIML-9-CRD1
MseIML-9-CRD2
MseIML-11-CRD1
MseIML-11-CRD2
MseIML-12-CRD1
MseIML-12-CRD2
MseIML-15-CRD2
MseIML-16-CRD2
MseIML-17-CRD1
MseIML-18-CRD1
MseIML-18-CRD2
MseIML-19-CRD1
MseIML-19-CRD2
MseIML-20-CRD1
MseIML-20-CRD2
MseIML-21-CRD1
MseIML-21-CRD2
MseIML-22-CRD1
MseIML-22-CRD2
MseIML-23-CRD1
MseIML-23-CRD2
MseIML-24-CRD2
MseIML-25-CRD2
MseIML-26-CRD1
MseIML-26-CRD2
MseIML-27-CRD1
MseIML-28-CRD1
MseIML-29-CRD1
Consensus
